# Delivery Mode Preference and Associated Factors among Pregnant Mothers in Harar Regional State, Eastern Ethiopia: A Cross-Sectional Study

**DOI:** 10.1155/2021/1751578

**Published:** 2021-05-10

**Authors:** Fissaha Tekulu Welay, Berhanu Gebresilassie, Guesh Gebreayezgi Asefa, Meresa Berwo Mengesha

**Affiliations:** ^1^Department of Midwifery, College of Medicine and Health Science, Adigrat University, Adigrat, Ethiopia; ^2^College of Health Science, Department of Biostatistics and Epidemiology, Aksum University, Ethiopia

## Abstract

**Background:**

The right to prefer mode of delivery is a crucial component of compassionate and respectful care that fosters both maternal and neonatal well-being as the failure to respect the mother's interest increases to the risk of maternal depression and posttraumatic stress. Thus, the aim of the study was to assess delivery mode preference and associated factors among pregnant women.

**Methods and Materials:**

The study was conducted in two hospitals and two health centers. We used a cross-sectional study design incorporating 398 pregnant mothers attending an antenatal care follow-up from February to May 2018. The study excluded pregnant mothers with any previous uterine surgery including caesarean delivery from participation due to their restricted chance to prefer their mode of delivery. Data were collected by using a pretested questionnaire. Data were entered to EpiData Manager version 3.1 and exported to Statistical Package for the Social Sciences version 22 for analysis. Besides, the analysis included both the bivariate and multivariable analyses to check the association between dependent and independent variables. Finally, level of statistical significance was declared at *P* value < 0.05.

**Result:**

The participant's level of response was 100% (398). The age of the mothers ranges from 15 to 45 years old. The delivery mode preference of the caesarean section (C/S) and spontaneous vertex delivery (SVD) was 115 (28.9%) and 283 (71.1%), respectively. The study revealed that planned 47 pregnancy [AOR, 1.76; CI: 0.89-3.47], young age [AOR, 12.9; CI: 0.23-7.1], and primigravida [AOR, 1.24; CI: 0.29-5.2] were among the variables associated with maternal preference of caesarean section. *Conclusion and Recommendation*. Nearly one-third of the mothers preferred caesarean delivery as their mode of delivery due to fear of labor pain and repeated vaginal examination by the care providers. This is particularly seen in women who had received higher education level, claim their pregnancy as planned, their choice of delivery at hospital, young aged, pregnant for the first time, and those who had visited antenatal care repeatedly. This implies that policy makers and stakeholders should exert due emphasis to ongoing desire of caesarean delivery as the procedure is not without risk, if it is done without indication. For researchers, we recommend to investigate the preference of mode of delivery in a much broader aspect.

## 1. Background

The right to choose mode of delivery is a crucial component of compassionate and respectful care in modern obstetrics as it fosters both maternal and neonatal well-being. Client-centered care is the core of health service delivery system involving client's decision-making about their treatment preference [1]. Hence, the pregnant mother's desire to prefer their mode of delivery is profound, as failure to respect their interest may expose the mother to depression and posttraumatic stress [2, 3]. Currently, on-demand caesarean section (C/S) appropriateness and ethical aspect is yet the point of debate for both obstetricians and women's groups. The questions about risks, benefits, and woman's autonomy of both C/S and SVD are the epicenter of the debate [4]. The growing attention in pregnant mothers to prefer childbirth options affects the medical and obstetrical decision [5]. Globally, there is a growing concern about the dramatically rising in the rates of caesarean section because of birth attendant failure to attempt a vaginal birth after a previous caesarean delivery (VBAC) [4]. However, this is not a sufficient reason to persuade researchers as primary caesarean delivery is also growing equally to the total caesarean delivery rate [6, 7]. Despite the World Health Organization (WHO) recommends a maximum of 10-15% acceptable rate of caesarean section in the presence of reasonable indications, many countries are reporting beyond this rate due to maternal preference [6]. For example in the United States, District of Colombia, the overall caesarean delivery rate was 32.8% in 2010, including 26.4% low-risk multiparous women.

Similarly, in Egypt, the rate of C/S almost doubled in four years as it increases from 27.6% in 2010 to 52% in 2014 [8, 9]. Besides, the reports from Addis Ababa, Ethiopia, in 2010 showed a considerable rate of increase from 2.3% to 24.4% within 14 years prior to the report. Moreover, researches from Iran and Ethiopia showed that maternal preference for their mode of delivery is associated with fear of labor pain, vaginal injury, influence on postpartum sexual life, and exposure to anesthesia [4, 10]. Although the incidence of caesarean delivery is growing abruptly, little is known whether the rise is due to maternal preference or providers failure to adhere the obstetrical indications and preforming the procedure at their own will to save the time spent by spontaneous vertex delivery (SVD) and there is a lack of clarity on the contributing factors of preference of mode of delivery in this context and to fully understanding its impacts. Additionally, assessing the pregnant mother's interest in relation to the obstetrical indication of both caesarean section (C/S) and vaginal delivery is a vibrant area of controversy that demands research [11]. Hence, the aim of the study was to determine the delivery mode preference and associated factors among ANC attendant pregnant mothers.

## 2. Methods and Materials

### 2.1. Study Area, Design, and Period

The study was conducted in two hospitals and two health centers found in the region using a cross-sectional study design. Three hundred and ninety-eight (398) pregnant mothers who attended the ANC follow-up from February to May 2018 participated in the study.

### 2.2. Sample Size Determination, Study Population, and Sampling Technique

The sample size was determined by using a single population proportion formula (*n* = (*Z*/2)2*pq*/*d*2), assuming confidence level at 95% = 1.96, a margin of error = 0.05, and proportion (*P*) = 37.8% of mothers prefer caesarean delivery according to the study conducted in Egypt [12]. This study included all ANC attendant pregnant mothers and excluded those who undergo any previous uterine surgery including caesarean delivery as mothers were restricted to prefer their delivery mode due to obstetric contraindication. The study implemented simple random sampling technique to select the study facilities included in the survey, whereas systematic sampling technique was used to filter the real respondents from their ANC registration frame.

### 2.3. Data Collection Tool and Methods

The authors developed the tool by incorporating different variables from different studies. It was first developed in English and translated into local language for the ease of data collection. The questionnaire was pretested on 5% of the total sample size out of the study area to prove its reliability, validity, and consistency ahead of the data collection. We have provided training to the data collectors about the objective of the study, method of data collection, and the tool. Finally, data were collected through a face-to-face interview by using pretested questionnaire immediately after the completion of antenatal care service provision.

### 2.4. Study Variables

The dependent (outcome) variable of the study was delivery mode preference (SVD vs. C/S) which had a dichotomy outcome, whereas the independent variables (predictors) included sociodemographic characteristics such as maternal age, maternal marital status, maternal educational, maternal occupation, Husband's educational status, and maternal residency.

Obstetric factors include parity, bad obstetric history, and pregnancy plan, and the reasons for SVD and the reasons for C/S mother's reproductive right-related factors include ANC follow-up, birth attendant, and place of delivery.

### 2.5. Data Processing, Analysis, and Ethics

The data clerk entered and cleaned the data using EpiData statistical software version 3.1. The authors exported the data set to SPSS statistical software version 22 for analysis. In addition to the descriptive statistics, the odds ratio was computed, to see the measure of the association between the independent and the outcome variables. Hence, variables with *P* value ≤ 0.25, in the binary logistic regression, undergo a multicollinearity test to prove the association among the independent variables. Thus, the variable with variance inflation factor (VIF) > 10 was dropped from the analysis, and the rest variables run in the multivariable regression model to control for possible confounding effect.

Besides, we have checked model fitness using the Hosmer-Lemeshow and omnibus tests. Thus, Pearson's chi-square was not statistically significant in the Hosmer-Lemeshow and found to be significant in the omnibus test. Finally, the results were presented in the form of texts, tables, and graphs with frequencies, measure of central tendency, and variability. The association between the different independent variables and delivery mode preference of pregnant mothers was presented in the form of a table with their crude odds ratio (COR) and adjusted odds ratio (AOR) along with their 95% confidence interval (CI).

Thus, the level of statistical significance was declared at *P* value < 0.05. Haramaya University Institutional Health Research Ethics Review Committee (IHRERC) permitted the study and approved it by a clearance letter. Informed, written, and signed consent was obtained from each participant after explaining the purpose and benefits of the study.

### 2.6. Definition of Terms

A spontaneous vertex delivery (SVD) is a vaginal delivery that happens on its own, without requiring doctors to use tools to help pull the baby out. This occurs after a pregnant woman goes through labor. Labor opens, or dilates, her cervix to at least 10 centimeters.

Caesarean section (C/S) is a surgical procedure used to deliver a baby through incisions in the abdomen and uterus.

Planned pregnancy means a purposeful thinking about what it means to have a baby and making decisions with their partner about their family.

## 3. Results

### 3.1. Participant's Sociodemographic Characteristics

The participant's level of response was 100% (398). The age of the mothers ranges from 15-45 years old with a mean (±SD) age of 26 (±11.34) years. The majority of the mothers were Muslim in religion, Oromo in ethnicity, and married. The husband of the married mothers educated to different level of education as discussed below (Table 1).

### 3.2. Obstetric Factors

Regarding the obstetric history, the gravidity of the mothers ranges from 1-8 conceptions with median (±SD) gravidity of 2 and S/D of (±1.17). The study revealed that 177 (44.5%) were primigravida, 204 (51.3%) multigravida, and 17 (4.3%) were grand multigravida mothers. During the study period, 51 (12.8%) were visiting ANC for the first time, while 102 (25.6%), 109 (27.4%), and 136 (34.2%) mothers were visiting for the 2^nd^, 3^rd^, and 4^th^ times, respectively. Besides, 325 (81.7%) mothers explained as they got counseling about the mode of delivery during their ANC follow-up while the rest 73 (18.3%) did not get anything about the issue.

### 3.3. Mother's Reproductive Right-Related Factors

The study revealed that 302 (75.9%) mothers explained as their pregnancy is planned while 96 (24.1%) mothers reported as it occurs accidentally. Regarding the birth assistant preference, 80 (20.1%) preferred to be assisted by doctor during birth, 22 (5.5%) by health officer, 263 (66.1%) by nurse/midwife, and 33 (8.3%) by health extension workers. Meanwhile, most of the participants (320) (80.4%) preferred to give birth in hospitals by female birth attendants and 78 (19.6%) in health centers by male birth attendants.

### 3.4. Delivery Mode Preference

**Figure 1:**
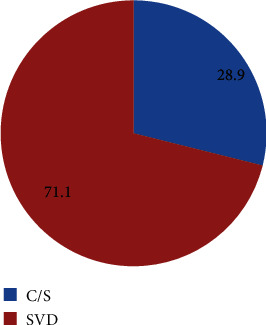
Mode of delivery preference among pregnant mothers in Harar Regional State, Eastern Ethiopia, 2018 (*N* = 398).

In this study, the pregnant mothers' preference for (C/S) and (SVD) as their mode of delivery was 115 (28.9% with 95% CI: 24.6%, 33.4%) and 283 (71.1% with 95% CI: 66.6%, 75.4%), respectively (Figure 1).

### 3.5. Reasons for a Mode of Delivery Preference

**Figure 2:**
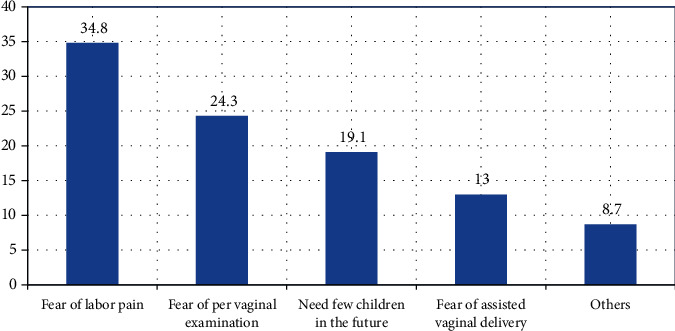
Reason for C/S delivery mode preference among pregnant mothers in Harar Regional State, Eastern Ethiopia, 2018 (*n* = 115). Others: fear of infection, surgical scar related to cosmetic reasons, and less neonatal asphyxia.

The study showed that most of the mothers (283) (71.1%) preferred to give birth through SVD due to certain reasons. Hence, the majority of the participants (120) (42.4%) mentioned fear of surgical operation, 80 (28.3%) need a short hospital stay, 74 (26.1%) had future fertility desire, and few mothers (9) (3.2%) list other reasons. Similarly, mothers preferred to give birth by C/S for certain reasons (Figure 2).

### 3.6. Factors Associated with Mode of Delivery Preference in the Bivariate and Multivariable Regression Model

Our study assessed some variables for their possible impact on maternal preference towards their mode of delivery. Thus, the mother's compliance with ANC visit increases the tendency of preferring C/S as their mode of delivery. Similarly, mothers with planned pregnancy [AOR = 1.76, 95%CI : 0.89, 3.47] were more likely to choose C/S than their counterparts (Table 2).

## 4. Discussion

Despite the obligatory measures taken by obstetricians to determine the mode of delivery using medical and obstetrical indications, pregnant mothers had reproductive right to prefer how and where to give birth. Besides, there is recent strong evidence that caesarean delivery showed increased maternal and neonatal infection. The aim of this study is therefore to determine maternal preference towards their mode of delivery and associated factors among pregnant mothers attending ANC visits. The result of this study found that nearly one-third of mothers preferred caesarean delivery as their mode of delivery.

This is almost consistent with the study conducted in Cerrahpasa Medical Faculty of Istanbul University where C/S and SVD preference was 32.8 and 67.14%, respectively [10]. The similarity might arise as both studies were conducted during ANC visit. However, our study result showed higher preference to C/S compared to other studies conducted in Sheffield, UK (21%) [13], university hospital of Assiut, Egypt (C/S = 20%) [14], six European countries (Belgium, Iceland, Denmark, Estonia, Norway, and Sweden) where preference to C/S was 12.2% [15], Hawassa City of Ethiopia (C/S = 13%) [16], and hospitals of Buenos Aires (C/S = 12%) [17]. The discrepancy emerged, as our study interviewed the mothers in ANC unit, whereas the reference studies conducted in delivery units which may decrease mothers tendency to choose C/S due to fear when labor approaches. Meanwhile, comparing to our result, much lower intention to C/S was observed in University of California (1.2%), in Nerchowk, Himachal Pradesh (7%), and in State of Pennsylvania (C/S = 3.1%) [18–20]. Hence, mothers were surveyed in the postpartum period after they undergo C/S for their next birth preference. This could enable them to choose SVD due to fear of current exposure to C/S. On the other hand, the preference of C/S as the mode of delivery is in our study is lower than the study revealed in Ismalia and Mina District of Egypt (C/S = 37.8% and 35.2%) [12, 21]. The increased preference for C/S in those studies is because the mothers were not pregnant at the time of the study. Hence, mothers do not give more emphasis to their mode of delivery preference and choose C/S due to arbitrary bias. Increased maternal educational status, young maternal age, and increased ANC follow-up were among the factors associated with C/S preference. This is in agreement with the study of Cerrahpasa Medical Faculty of Istanbul University and six European countries, as education increases awareness of mothers about their health and adherence to ANC visit increase the chance of being counseled about the mode of delivery. Besides, mothers had planned pregnancy, educated husband, and lower gravid status increase the preference of caesarean delivery. This study disproves the association between urban residency and occupational status with C/S preference unlike explained in Cerrahpasa Medical Faculty and Hawassa City of Ethiopia [10, 16]. The findings of this study implies that although maternal preference does not necessarily signify increase in C/S due to obstetrician decision, the desire is higher than WHO maximum C/S rate. Understanding how pregnancy outcomes are affected by mode of delivery is important to ensure that the intervention would be safe or hazard for both the mother and neonate. Caesarean sections performed for appropriate medical or obstetric indications are life saving for both the mother and the newborn. But the high prevalence of caesarean section is not associated with improved perinatal outcome and it has risks for the mother and the neonate. Therefore, this finding could have an importance to see our present practices towards preference of mode of delivery.

## 5. Conclusion and Recommendations

Generally, a significant number of mothers involved in the study preferred C/S as their mode of delivery. The main reasons for the preference were fear of labor pain and repeated vaginal exam during SVD.

The higher C/S preference is observed in mothers who wanted to give birth in the hospital, had planned pregnancy, educated, young aged, lower gravid status, and those with increased ANC visits.

Although, maternal preference does not necessarily signify increase in C/S due to obstetrician decision, the desire is higher than WHO maximum C/S recommendation. Thus, policy makers and stakeholders should exert due emphasis to ongoing desire of C/S delivery as the procedure is not without risk. We recommend other researchers to investigate the preference of mode of delivery in much broader aspect.

## 6. Limitations of the Study

We notice two drawbacks in this research. First, this study excluded of mothers who had previous uterine rupture or C/S due to restricted preference to choose their mode of delivery by obstetric indications. Second, the study design implemented (cross-sectional study design) is weak to show the temporal relationship between real causes (factors) to determine C/S preference among pregnant mothers.

## Figures and Tables

**Table 1:** Participant's sociodemographic characteristics among pregnant mothers in Harar Regional State, Eastern Ethiopia, 2018.

Variables	Delivery mode preference		Total/percent (%)
	C/S	SVD	
Religion (*N* = 398)			
Muslim	37	154	182 (45.7%)
Orthodox	56	78	134 (33.7%)
Catholic	6	21	27 (6.8%)
Protestants	16	39	55 (13.8%)
Ethnicity (*N* = 398)			
Oromo	39	150	189 (47.5%)
Amhara	53	72	125 (31.4%)
Harar	7	21	28 (7%)
Gurage	10	19	29 (7.3%)
Others	6	21	27 (6.8%)
Maternal educational status (*N* = 398)			
Yes	94	226	320 (80.4%)
No	21	57	78 (19.6%)
Maternal marital status (*N* = 398)			
Married	114	279	393 (98.7%)
In relationship	1	4	5 (1.3%)
Husband/partner/educational status (*N* = 398)			
Read and write	18	47	65 (16.3%)
Primary level	38	111	149 (37.4%)
Secondary level	26	69	95 (23.9%)
College and above	33	56	89 (22.4%)
Maternal age (*N* = 398)			
<18 years	1	6	7 (1.8%)
18-30 years	96	254	250 (87.9%)
>30 years	18	23	41 (10.3%)
Maternal residence (*N* = 398)			
Urban	114	260	374 (94%)
Rural	1	23	24 (6%)

C/S: caesarean section; SVD: spontaneous vertex delivery; *N*: number.

**(a):** 

Variable	Frequency (%)	Delivery mode preference		COR (95% CI)^∗^	*P* value	AOR (95% CI)^∗∗^	*P* value
		C/S	SVD				
Maternal level of education							
Read and write	41 (12.5%)	16	25	1.00		1.00	
Primary level	114 (35.6%)	21	93	1.08 (0.5, 2.3)	0.048	0.9 (0.4, 2.01)	
Secondary level	77 (24.1%)	25	52	3.06 (1.62, 5.79)	0.002	2.6 (1.35, 5.06)^∗∗^	0.03
College and above	88 (27.1%)	36	52	1.4 (0.76, 2.73)		1.3 (0.7, 2.68)	
Husbands' education							
Read and write	65 (16.3%)	18	47	1.00		1.00	
Primary level	149 (37.4%)	38	111	1.54 (0.77, 3.07)		1.8 (0.8, 4.01)	
Secondary level	95 (23.9%)	26	69	1.72 (0.97, 3.03)		1.9 (0.97, 3.6)	
College and above	89 (22.4%)	33	56	1.56 (0.83, 2.9)	0.004	1.78 (0.86, 3.6)	
Planned pregnancy							
Yes	302 (75.9%)	82	220	1.4 (0.86-2.29)	0.0018	1.76 (0.89, 3.47)^∗∗^	0.038
No	96 (24.1%)	33	63	1.00		1.00	
Time of ANC visit							
1^st^ visit	51 (12.8%)	13	38	1.00		1.00	
2^nd^ visit	102 (25.6%)	26	76	1.64 (0.8, 3.38)		1.36 (0.59, 3.13)	
3^rd^ visit	109 (27.4%)	27	82	1.64 (0.93, 2.9)	0.041	1.59 (0.82, 3.09)	
4^th^ visit	136 (34.2%)	49	87	1.7 (0.98, 2.98)	0.0031	1.66 (0.86, 3.17)^∗∗^	0.039
Age							
<18	7 (1.8%)	1	6	1.00		1.00	
18-30	350 (87.9%)	96	254	4.69 (1.52, 8.59)	0.023	2.9 (0.23, 7.1)^∗∗^	0.047
30-45	41 (10.3%)	18	23	2.07 (1.07, 4)		1.93 (0.86,4.3)	
Counseling on delivery mode							
Yes	325 (81.7%)	98	227	1.13 (0.41, 3.07)	0.026	0.7 (0.39, 1.27)	
No	73 (18.3%)	17	56	1.00		100	
Gravidity							
Primigravida	177 (44.5%)	51	126	1.34 (0.47, 3.83)	0.001	1.24 (0.29, 5.2)^∗∗^	0.029
Multigravida	204 (51.3%)	58	146	1.37 (0.48, 3.9)		1.23 (0.29-4.9)	
Grand multigravida	17 (4.3%)	6	11	1.00		1.00	
Birth place preference							
Hospital	320 (80.4%)	105	215	3.23 (1.9, 6.4)	0.0034	2.2 (1.5, 4.7)^∗∗^	0.025
Health center	60 (15.1%)	9	51	1.3 (0.87, 2.6)		1.00	

**(b):** 

Variable	Frequency (%)	Delivery mode preference		COR (95% CI)^∗^	AOR (95% CI)^∗∗^
		C/S	SVD		
Maternal level of education					
Read and write	41 (12.5%)	16	25	1.00	1.00
Primary level	114 (35.6%)	21	93	1.08 (0.5, 2.3)	0.9 (0.4, 2.01)
Secondary level	77 (24.1%)	25	52	3.06 (1.62, 5.79)	2.6 (1.35, 5.06)
College and above	88 (27.1%)	36	52	1.4 (0.76, 2.73)	1.3 (0.7, 2.68)
Husbands' education					
Read and write	65 (16.3%)	18	47	1.00	1.00
Primary level	149 (37.4%)	38	111	1.54 (0.77, 3.07)	1.8 (0.8, 4.01)
Secondary level	95 (23.9%)	26	69	1.72 (0.97, 3.03)	1.9 (0.97, 3.6)
College and above	89 (22.4%)	33	56	1.56 (0.83, 2.9)	1.78 (0.86, 3.6)
Planned pregnancy					
Yes	302 (75.9%)	82	220	1.4 (0.86-2.29)	1.76 (0.89, 3.47)
No	96 (24.1%)	33	63	1.00	1.00
Time of ANC visit					
1^st^ visit	51 (12.8%)	13	38	1.00	1.00
2^nd^ visit	102 (25.6%)	26	76	1.64 (0.8, 3.38)	1.36 (0.59, 3.13)
3^rd^ visit	109 (27.4%)	27	82	1.64 (0.93, 2.9)	1.59 (0.82, 3.09)
4^th^ visit	136 (34.2%)	49	87	1.7 (0.98, 2.98)	1.66 (0.86, 3.17)
Age					
<18	7 (1.8%)	1	6	1.00	1.00
18-30	350 (87.9%)	96	254	4.69 (1.52, 8.59)	2.9 (0.23, 7.1)
30-45	41 (10.3%)	18	23	2.07 (1.07, 4)	1.93 (0.86, 4.3)
Counseling on delivery mode					
Yes	325 (81.7%)	98	227	1.13 (0.41, 3.07)	0.7 (0.39, 1.27)
No	73 (18.3%)	17	56	1.00	100
Gravidity					
Primigravida	177 (44.5%)	51	126	1.34 (0.47, 3.83)	1.24 (0.29, 5.2)
Multigravida	204 (51.3%)	58	146	1.37 (0.48, 3.9)	1.23 (0.29-4.9)
Grand multigravida	17 (4.3%)	6	11	1.00	1.00
Birth place preference					
Hospital	320 (80.4%)	105	215	3.23 (1.9, 6.4)	2.2 (1.5, 4.7)
Health center	60 (15.1%)	9	51	1.3 (0.87, 2.6)	—
Private clinic	18 (4.5%)	1	17	1.00	1.00

^∗^Significant at *P* value of 0.25. ^∗∗^Statistically significant at *P* value of 0.05. CI: confidence interval; AOR: adjusted odds ratio; COR: crude odds ratio; ANC: antenatal care.

## Data Availability

The datasets used and/or analyzed during this study are available from the corresponding author on reasonable request.

## Abbreviations

ANC: Antenatal care
AOR: Adjusted odds ratio
COR: Crude odds ratio
CI: Confidence interval
IHRERC: Haramaya University Institutional Health Research Ethics Review Committee
C/S: Caesarean section
SPSS: Statistical Package for the Social Sciences
SVD: Spontaneous vaginal delivery
VBAC: Vaginal birth after caesarean section
VIF: Variance inflation factor
WHO: World Health Organization.
